# Diagnostic Significance of Diffusion-Weighted MRI in Renal Cancer

**DOI:** 10.1155/2015/172165

**Published:** 2015-04-30

**Authors:** Yu Lei, Hong Wang, Hai-Feng Li, Yan-Wei Rao, Jing-Hong Liu, Shi-Feng Tian, Ye Ju, Ye Li, An-Liang Chen, Li-Hua Chen, Ai-Lian Liu, Ming-Li Sun

**Affiliations:** Department of Emergency, The First Affiliated Hospital of Jilin University, Changchun, Jilin 130021, China

## Abstract

*Background.* This study aimed to investigate whether diffusion-weighted imaging (DWI) could contribute to the discrimination between benign and malignant renal cancer. *Methods.* We searched the PubMed electronic database for eligible studies. STATA 12.0 software was used for statistical analysis. The SMD and 95% CI were calculated. *Results.* Decreased ADC signal was seen in all renal cancer patients (cancer tissue versus normal tissue: SMD = 1.63 and 95% CI = 0.96~2.29, *P* < 0.001; cancer tissue versus benign tissue: SMD = 2.22 and 95% CI = 1.53~2.90 and *P* < 0.001, resp.). MRI machine type-stratified analysis showed that decreased ADC signal was found by all included MRI machine types in cancer tissues compared with benign cancer tissues (all *P* < 0.05). The ADC values of renal cancer patients were significantly lower than those of normal controls for all included *P* values (all *P* < 0.05), and there was a decreased ADC signal at *b*-500, *b*-600, *b*-1000, *b*-500, and 1000 gradients compared with benign cancer tissues (all *P* < 0.05). *Conclusion.* Our study concluded that decreased ADC signal presented in DWI may be essential for the differential diagnosis of renal cancer.

## 1. Introduction

Renal cancer is a metabolic disease that starts in the cells in the kidney, consisting of a number of different types of cancer, and the commonest type is renal cell carcinoma (RCC) which accounts for approximately 90% of all renal cancers [1–3]. Epidemiological evidence supported the fact that renal cancer ranks for the 13th most common cancer in the world, with about 270,000 new cases diagnosed annually, and 116,000 people die from the disease [4]. In addition, the most common presenting symptoms of renal cancer are as follows: flank and back pain, fatigue, anaemia, haematuria, weight loss, and so forth [5, 6]. Furthermore, the risk of renal cancer in men is investigated to be about two times higher than that in women [4]. Although the etiology of renal cancer is poorly understood, interaction between several environmental and genetic factors could influence the risk of developing renal cancer [7]. Cigarette smoking, obesity, and hypertension are considered to be causal risk factors for renal cancer [8–10]. Currently, renal masses can be detected and characterized by using ultrasound (US), computed tomography (CT), or magnetic resonance imaging (MRI) [11]. However, there is consensus that MRI diffusion-weighted imaging technique plays a more important role in the differential diagnosis of benign and malignant renal tumors [12–14].

Diffusion-weighted imaging (DWI) evaluates random movement of water molecular diffusion process in vivo, which can provide information on the spatial structure and biophysical characteristics of tissues such as cellular structure, cellular density, microstructure, and microcirculation [15, 16]. Lesions with dense cytoarchitectonics and poor interstitial spaces that restrict the microscopic mobility of water molecules within and between the intracellular and extracellular spaces exhibit high or bright signal intensity on DWI, which has been applied to the diagnosis of malignancy [17, 18]. In general, most neoplasms show restricted diffusion owing to the dense cytoarchitectonics of solid tumors and increased cell membranes per unit volume, leading to the restriction of water molecular movement and corresponding high signal intensity on DWI [19]. The degree of water molecules diffusion can be evaluated quantitatively by the apparent diffusion coefficient (ADC) value [20]. As a quantitative parameter calculated from the DWI images, the ADC value can reflect the pathological changes of tissues and is very useful in the clinical diagnosis of central nervous system diseases, various abdominal lesions, and especially renal diseases [21, 22]. The ADC value is inversely proportional to cellular density because increased cellular density limits water diffusion in the interstitial space [23]. In the past few decades, a large body of evidence has suggested that DWI with quantitative ADC measurements can act as a predictor in differentiating malignant renal lesions from normal kidney and benign renal lesions [24, 25], whereas other studies have arrived at different findings [26, 27]. Given the conflicting evidence on this issue, we performed a pooled analysis to evaluate the diagnostic value of DWI and the ADC value in differentiating malignant renal tumors from benign renal cancers.

## 2. Materials and Methods

### 2.1. Search Strategy

We searched for relevant published studies in PubMed electronic database from their inception until April 2014. The searching was performed using “Carcinoma, Renal Cell” and “Diffusion Magnetic Resonance Imaging” as the Medical Subject Headings (MeSH) evaluating the DWI in discriminating between benign and malignant renal cancers and corresponding to the following free text word searching terms: (“renal carcinoma” or “kidney carcinoma” or “kidney cancer” or “renal neoplasms” or “kidney neoplasms” or “kidney tumor” or “Renal Cell Cancer” or “RCC” or “renal cell carcinoma”) and (“Diffusion MRI” or “Diffusion Weighted MRI” or “Diffusion Magnetic Resonance Imaging” or “DWI” or “diffusion-weighted magnetic resonance imaging” or “MRI-DWI” or “diffusion-weighted imaging” or “diffusion-weighted-MRI”). There was no language restriction used in the search strategy. We also searched the reference lists of pertinent articles.

### 2.2. Selection Criteria

To be included in the analysis, these studies must be in accordance with the following criteria: (1) they are clinical case-control studies, cohort studies, cross-sectional studies, or randomized controlled trials; (2) all patients diagnosed with renal cancer must be confirmed by histopathologic examinations; (3) accuracy of MRI must be evaluated in differential diagnosis between benign and malignant renal cancer; (4) sufficient information must be provided within the study about the criteria for evaluating the levels of DWI or ADC. Studies were excluded if they did not meet all the above inclusion criteria. When more than one study by the same author using the same case series was published, the study with either the most recent publication or the largest sample size was included to avoid overlapping populations. Any disagreements were resolved through discussions and subsequent consensus.

### 2.3. Data Extraction

Using a standardized form, two authors independently extracted data from eligible studies. The extracted data included the characteristics of the subjects such as age, sex, and other treatment, as well as the study design, year of publication, source of publication, country of origin, ethnicity, language of publication, study type, total number of subjects or samples, source of subjects or samples, pathological subtype, number of lesions, MRI machine type, contrast agent, and diagnostic accuracy. Study authors were contacted as needed to obtain detailed data. In cases of conflicting evaluations, any disagreements were resolved by a consensus among the investigators.

### 2.4. Quality Assessment

The included articles were summarized both qualitatively and quantitatively. The quality of those included studies was assessed independently by two investigators based on a tool for the quality assessment of studies of diagnostic accuracy studies (QUADAS) [28]. Fourteen assessment items were implicated in these QUADAS criteria. Each of these items was scored as “yes” (2), “no” (0), or “unclear” (1). QUADAS score ranged from 0 to 28, and scores ≥ 22 indicate a good quality. Disagreements on the quality assessments of the included studies were resolved through a comprehensive reassessment by the authors.

### 2.5. Statistical Analysis

All analyses were calculated using the STATA software, version 12.0 (Stata Corp., College Station, TX, USA). In this pooled analysis, the standardized mean difference (SMD) was combined with the 95% confidence intervals (CIs) calculated using the random-effect model. The significance of the pooled estimate was made using the *Z* test. We estimated the degree of heterogeneity among studies using Cochran's *Q*-statistic, which is regarded as significant at *P* < 0.05 [29]. Heterogeneity among the studies was evaluated using the *I* ^2^ test (ranges from 0 to 100%) [30]. When a significant *Q*-test with *P* < 0.05 or *I* ^2^ > 50%, was observed, the random-effect model (DerSimonian Laird method) was then used. Nevertheless, when there was no statistical heterogeneity, we used a fixed-effects model (Mantel-Haenszel method). For the purpose of exploring potential sources of heterogeneity, subgroup analyses were performed based on ethnicity and MRI machine type. We conducted a sensitivity analysis by omitting each study to evaluate the influence of single studies on overall estimate. The possibility of a publication bias, which can result from the nonpublication of small studies with negative findings, was assessed visually using a funnel plot for asymmetry. The symmetry of the funnel plot was further evaluated by Egger's linear regression test [31]. All tests were two-sided and a *P* value of < 0.05 was considered statistically significant.

## 3. Results

### 3.1. Baseline Characteristics of Included Studies

A highly sensitive search strategy for identifying reports of cohort studies in electronic databases was performed. The electronic database search initially retrieved 98 studies. The studies were eliminated for being duplicates (*n* = 1), letters, reviews, or meta-analyses (*n* = 11), not human studies (*n* = 14), not related to research topics (*n* = 18), not case-control study (*n* = 8), not relevant to MRI-DWI (*n* = 12), and not relevant to renal tumors (*n* = 15) and having insufficient information or weakly correlated data (*n* = 2). Eventually, 16 clinical cohort studies with a total of 1,428 renal cancer patients were enrolled in the study for quantitative data analysis [13, 14, 18, 20, 22, 24–27, 32–38]. Publication years of the eligible studies ranged from 2004 to 2013. Overall, 8 studies were among Caucasians, another 7 studies were among Asians, and the remaining one was among Africans. Different kinds of MRI machines were chosen in those articles, such as GE 3.0 T, Tesla 1.5 T, Siemens 1.5 T, Philips 1.5 T, GE 1.5 T, and Philips 3.0 T. QUADAS scores of all included studies were ≥20. We summarized the study characteristics and methodological quality in Table 1.

### 3.2. Quantitative Data Synthesis

**Figure 1:**
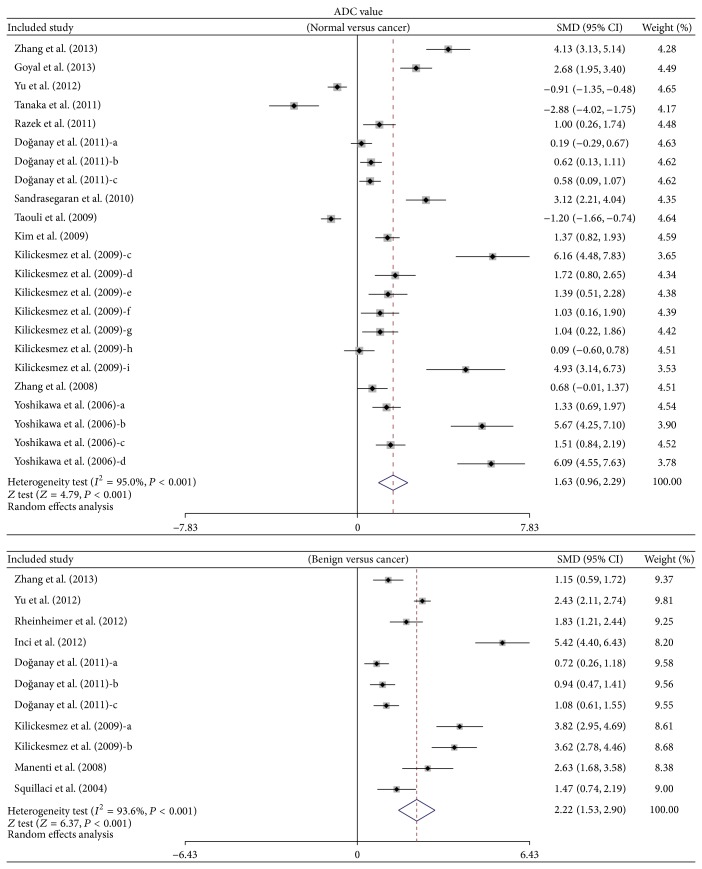
Forest plots on the difference in the frequency of ADC value between cancer tissues and benign tissues in renal cancer patients.

A total of sixteen studies were included to assess the potential role of DWI in distinguishing malignant renal cancer from benign renal cancer. The random-effects model was used since heterogeneity was significantly observed. In the pooled estimation, our findings demonstrated that decreased ADC signal was seen in all our renal cancer patients when compared to the healthy subjects (cancer tissue versus normal tissue: SMD = 1.63 and 95% CI = 0.96~2.29, *P* < 0.001). A similar result was also discovered with regard to the decline of ADC signal in cancer patients when compared with those of benign patients (cancer tissue versus benign tissue: SMD = 2.22 and 95% CI = 1.53~2.90, *P* < 0.001) (Figure 1).

**Figure 2:**
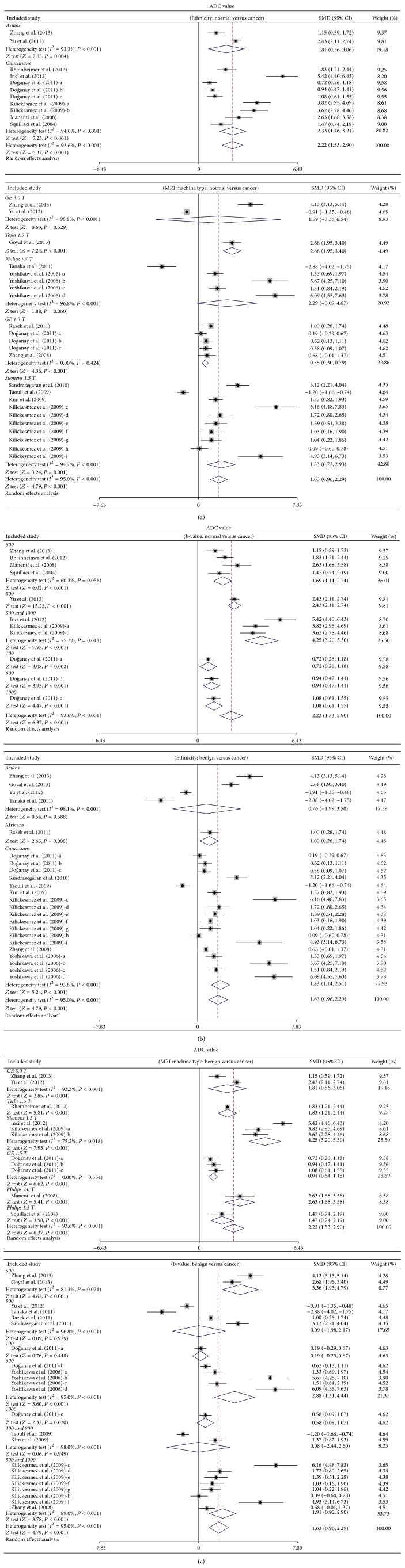
Subgroup analyses by ethnicity and MRI machine type on the difference of ADC value between cancer tissues and benign tissues in renal cancer patients.

Further subgroup analysis was undertaken to evaluate the value of the ADC signal in the discrimination of malignant and benign renal cancer. In the ethnicity subgroup analysis, the decreased signal of ADC was statistically significant in cancer cases among the Asians and Caucasians in comparison to normal tissue (Asians: SMD = 1.81 and 95% CI = 0.56~3.06, *P* = 0.004; Caucasians: SMD = 2.33 and 95% CI = 1.46~3.21, *P* < 0.001, resp.). The decreased signal of ADC in the cancer tissue was also found among African and Caucasian populations when compared with the benign tissue (Africans: SMD = 1.00 and 95% CI = 0.26~1.74, *P* = 0.008; Caucasians: SMD = 1.83 and 95% CI = 1.14~2.51, *P* < 0.001, resp.) (Figure 2). In addition, in the stratified analysis based on MRI machine type, we also observed an association of the decreased signal of ADC in renal cancer patients among GE 1.5 T, Tesla 1.5 T, and Siemens 1.5 T types (all *P* < 0.05), whereas no such result was observed in the GE 3.0 T and Philips 1.5 T as compared with normal healthy controls (GE 3.0 T: SMD = 1.59 and 95% CI = −3.36~6.54, *P* = 0.529; Philips 1.5 T: SMD = 2.29 and 95% CI = −0.09~4.67, *P* = 0.060, resp.). On the other hand, when compared with benign renal cancer patients, the ADC signal was revealed to be decreased in all the experimental MRI machine types in the malignant renal cancer patients (all *P* < 0.05) (Figure 2). The findings of the subgroup analysis by *b*-value illustrated that ADC values of renal cancer patients were significantly lower than those of normal controls in all included *b*-values (all *P* < 0.05), with a decreased ADC signal at *b*-500, *b*-600, *b*-1000, *b*-500 & 1000 gradients compared with benign cancer tissues (all *P* < 0.05).

**Figure 3:**
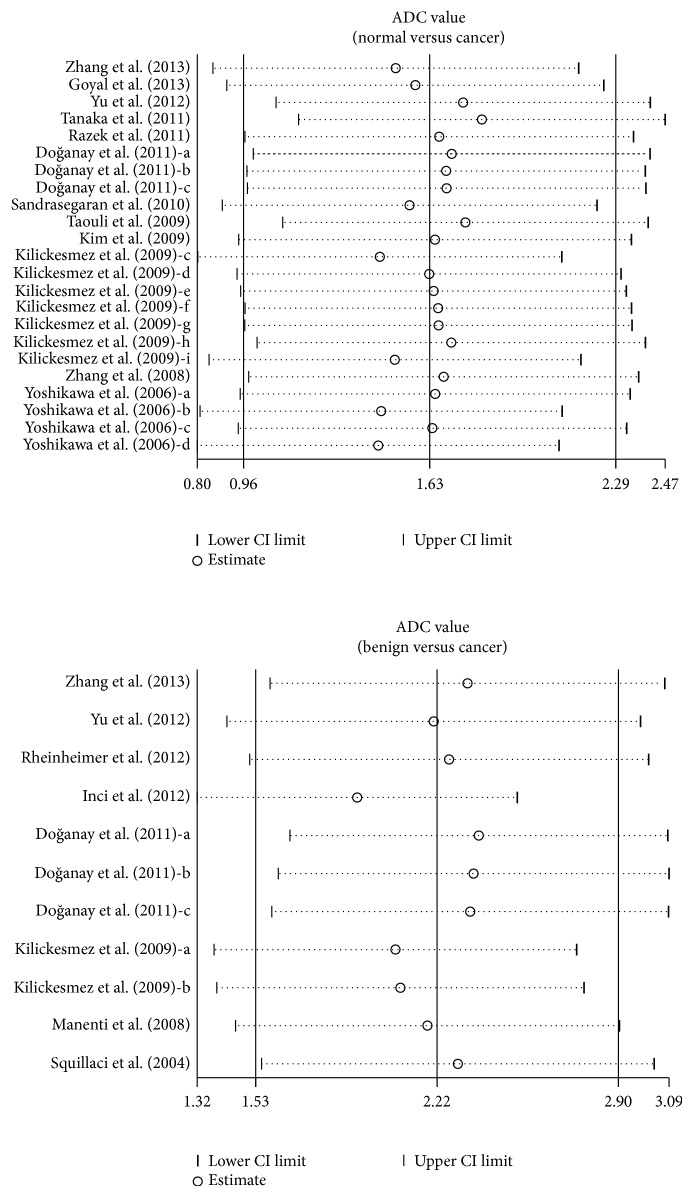
Sensitivity analysis of the summary odds ratio coefficients on the difference in the frequency of ADC value between cancer tissues and benign tissues in renal cancer patients.

**Figure 4:**
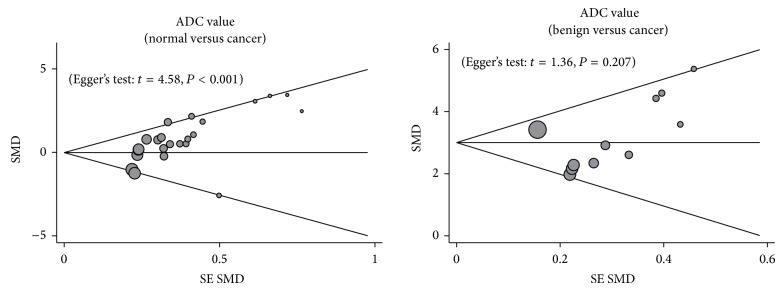
Funnel plot of publication biases on the difference in the frequency of ADC value between cancer tissues and benign tissues in renal cancer patients.

By ignoring individual studies in turn, we carried out a sensitivity analysis to assess the effects of each individual study on the pooled estimates, with results indicating that no single study could influence the overall pooled estimates (Figure 3). The funnel plots showed obvious asymmetry in the observation of ADC signal in the normal versus cancer model, and Egger's test also presented strong evidence of publication bias (*t* = 4.58, *P* < 0.001), while under the benign versus cancer model, funnel plots presented no obvious asymmetry, and Egger's test also showed no evidence of publication bias (*P* > 0.05) (Figure 4).

## 4. Discussion

Diffusion imaging is based on the natural sensitivity of MR to motion, and diffusion-weighted images are obtained by incorporating strong magnetic field gradient pulses within any imaging pulse sequence [39]. Diffusion MR imaging techniques are increasingly varied recently, from the simplest and most commonly used technique, the mapping of ADC values, to the more complex, such as diffusion tensor imaging, diffusion spectrum imaging, Q-ball imaging, and tractography [40]. The present pooled analysis was performed to explore the diagnostic value of DWI in differentiating malignant renal tumors from normal renal tissues and benign renal diseases via measuring the ADC values of these lesions. In this pooled analysis, the findings revealed that the ADC values of malignant renal tumors were significantly lower than those of normal renal tissues and benign renal diseases, implying that DWI with quantitative ADC measurements may play a crucial role in diagnosis of malignant renal lesions from benign renal diseases. As we all know, water molecules movement plays an important role in the kidney functions including reabsorption, concentration, and dilution of urine [41]. The technology of DWI adopts DW gradient pulses to produce signals which are susceptible to the localized diffusivity of water molecules and thus can indirectly measure the renal cell density [42]. Renal tissues with different cellular structure, such as renal parenchyma structure and neoplastic tissue anarchic structure, may display different ADC values on DWI, which can provide information for recognizing and characterizing renal masses [32]. Consequently, DWI with ADC values can be helpful methods in the diagnosis and quantitative measurement of neoplasms. Many studies have showed that increased or higher signal intensity was seen on DWI and decreased signal on ADC maps of most malignant tumors when compared to the benign lesions and normal tissues [12, 16, 43]. The precise mechanisms of malignant renal tumors having lower ADC values are still unclear, but it is maybe associated with a combination of dense cytoarchitectonics and poor interstitial spaces in malignant cells, which may restrict the random movement of water molecules [13]. In line with our results, a previous study has also demonstrated that renal tumors with dense cell architecture have higher ADC value, which can differentiate benign from malignant renal tumors, suggesting that elevated ADC value may be a useful method in clinical diagnosis of renal cancer [36]. To be consistent with the present study, a study conducted by Inci et al. has showed that malignant renal tumors had significant lower ADC values in contrast with benign diseases, and DWI can be considered to be a useful investigative tool for diagnosing, characterizing, and staging renal masses, which can contribute additional value by promising differentiation of benign from malignant renal tumors [25]. In addition, DWI acquisition can usually be challenging in the abdomen due to the breathing related motion artifacts. Although this does restrict the accurate data collection of DWI and other confounding factors cannot be completely ruled out, the clinical application of DWI to oncology, which includes gastric cancer, has become considerably more frequent, since qualitative and quantitative information regarding high cellular tumor-tissue and water molecules differences in diffusion can be obtained [44, 45].

To investigate the influence of potential factors on the diagnostic value of DWI in differentiating malignant renal tumors from normal renal tissues and benign renal diseases, we carefully performed stratified analyses based on ethnicity and MRI machine type. Our results showed that the ADC values of normal renal tissues on DWI were obviously higher than those of malignant renal tumors on DWI among Asians and Caucasians. Furthermore, the ADC values of benign renal diseases on DWI were obviously higher than those of malignant renal tumors on DWI among Africans and Caucasians. Thus, this result suggested that ethnic differences may be a potential source of heterogeneity. In addition, further subgroup analysis performed by MRI machine type revealed that the ADC values of malignant renal tumors on DWI with most MRI machine types were significantly lower than those of normal renal tissues and benign renal diseases, whereas no such observation was detected from the results of the 3.0 T DWI machine. All in all, our results are in line with previous studies that DWI with quantitative ADC measurements can be considered a useful and noninvasive biological marker in differentiating renal cancer from normal tissues and benign renal diseases and can be used as an effective imaging method for tumor diagnosis.

Our analysis should be interpreted in the context of the following limitations. Firstly, we did not take into account unpublished articles and abstracts due to the restriction of inclusion criteria, and thus all relevant data may not have been obtained. In this regard, our results did not include all the data from all trials evaluating the relationship of ADC value with the differential diagnosis of renal cancer. A second limitation of this pooled analysis is that, due to the nature of pooled analysis, our results may be influenced by publication bias, especially since we only enrolled eligible English studies and thus may have excluded otherwise qualified studies based on language criteria. Thirdly, our investigation from those included sixteen articles did not take into consideration the cohort design, which also affects the DWI signal and therefore the ADC values; hence ADC values detected in the present trails may not be so reliable contributing to the final conclusion. Finally, usual reliable statistical packages (STATA) are only able to calculate unweighted kappa coefficients for multiple raters, where they are inappropriate for ordinal scales for their treatment of all disagreements equally. Despite the above limitations, this is the first example of pooled analysis on the association of DWI imaging analysis and ADC values with the development of GC. More importantly, all the included articles were conducted among all three populations, and a statistical approach was also adopted to combine the results from multiple studies. Besides, strict inclusion and exclusion criteria were carried out in selecting articles, whose inconsistent results were rigorously quantified and analyzed in this pooled analysis, leading to a more complete elucidation of this issue.

In conclusion, this pooled analysis supports the idea that decreased ADC signals are beneficial in differentiating between benign renal cancers and malignant renal cancers. The DWI imaging investigation may be considered as one essential method regarding the differential diagnosis of renal cancers. However, DWI acquisition still has drawbacks, such as the challenge of breathing related motion artifacts. Meanwhile, the findings of our pooled analysis underscore the need for long-term randomized prospective studies to confirm our findings.

## Figures and Tables

**Table 1:** Characteristics of included studies in this *pooled analysis*.

First author	Year	Ethnicity	Number			Gender (F/M)	Age (years)	MRI machine type	QUADAS score
			Tumor	Benign	Normal				
Zhang [27]	2013	Asians	45	12	20	31/13	52	GE 3.0 T	26
Goyal [26]	2013	Asians	40	20	0	26/10	46 (21~80)	Tesla 1.5 T	26
Yu [22]	2012	Asians	137	0	137	93/44	53 (30~81)	GE 3.0 T	28
Rheinheimer [38]	2012	Caucasians	28	0	30	17/9	62.0 ± 13.0	Tesla 1.5 T	22
Inci [25]	2012	Caucasians	42	0	30	59/46	55	Siemens 1.5 T	26
Tanaka [37]	2011	Asians	36	5	41	21/14	57 (38~78)	Philips 1.5 T	23
Razek [36]	2011	Africans	45	9	0	24/28	5~67	GE 1.5 T	26
Doğanay [14]	2011	Caucasians	32	35	50	25/33	53.0 ± 19.0	GE 1.5 T	25
Sandrasegaran [13]	2010	Caucasians	22	20	0	—	—	Siemens 1.5 T	22
Taouli [35]	2009	Caucasians	28	81	0	46/18	61 (24~85)	Siemens 1.5 T	24
Kim [34]	2009	Caucasians	26	38	0	—	—	Siemens 1.5 T	24
Kilickesmez [24]	2009	Caucasians	16	0	50	25/27	52	Siemens 1.5 T	20
				17	0				
				10	0				
				9	0				
				11	0				
				16	0				
				6	0				
Zhang [33]	2008	Caucasians	26	11	0				23
Manenti [18]	2008	Caucasians	27	0	10	16/11	62 (45~85)	Philips 3.0 T	21
Yoshikawa [20]	2006	Caucasians	28	19	0	—	—	Philips 1.5 T	20
			28	13	0	—	—	Philips 1.5 T	
			26	19	0	—	—	Philips 1.5 T	
			26	13	0	—	—	Philips 1.5 T	
Squillaci [32]	2004	Caucasians	18	0	20	10/8	62 (29~85)	Philips 1.5 T	20

M: male; F: female; QUADAS: quality assessment of diagnostic accuracy studies.
